# Decreased *Neisseria gonorrhoeae* genotypic diversity following COVID-19 restrictions in Queensland, Australia 2020

**DOI:** 10.1017/S0950268823000523

**Published:** 2023-04-13

**Authors:** Bushra Alharbi, Amy V. Jennison, Vicki Hicks, David M. Whiley, Emma Sweeney, Ella Trembizki

**Affiliations:** 1 Faculty of Medicine, Centre for Clinical Research, The University of Queensland, Brisbane, QLD, Australia; 2 Faculty of Pharmacy, Taibah University, Madinah, Saudi Arabia; 3 Public Health Microbiology, Queensland Health Forensic and Scientific Services, Brisbane, QLD, Australia; 4 Pathology Queensland Central Laboratory, Brisbane, QLD, Australia

**Keywords:** Antimicrobial resistance, COVID-19, genotyping, *Neisseria gonorrhoeae*, Sexually transmitted infections, Epidemiology

## Abstract

We investigated the potential effects of COVID-19 public health restrictions on the prevalence and distribution of *Neisseria gonorrhoeae* (NG) genotypes in our Queensland isolate population in the first half of the year 2020. A total of 763 NG isolates were genotyped to examine gonococcal strain distribution and prevalence for the first 6 months of 2020, with 1 January 2020 to 31 March 2020 classified as ‘pre’ COVID-19 restrictions (*n* = 463) and 1 April 2020 to 30 June 2020 classified as ‘post’ COVID-19 restrictions (*n* = 300). Genotypes most prevalent ‘pre’ restrictions remained proportionally high ‘post’ restrictions, with some significantly increasing ‘post’ restrictions. However, genotype diversity was significantly reduced ‘post’ restrictions. Overall, it seems public health restrictions (9–10 weeks) were not sufficient to affect rates of infection or reduce the prevalence of well-established genotypes in our population, potentially due to reduced access to services or health-seeking behaviours.

*Neisseria gonorrhoeae* (NG) rates are on the rise globally and rising gonococcal antimicrobial resistance (AMR) remains an urgent threat [1]. Understanding the factors that impact NG rates and transmission is important for informing public health interventions. In Queensland, Australia, rates of gonococcal infections have continued to steadily increase over the last two decades (like many regions locally and globally), with 5,058, 4,906, and 5,977 notifications in the years 2017–2019, respectively. To our surprise, despite stringent COVID-19 public health restrictions, infections again increased in 2020 to 6,346 [2, 3]. A further breakdown of 2020 showed that rates remained somewhat consistent throughout the year’s quarters (see Supplementary Table S1) as well as comparable to previous years (see Supplementary Figure S1). On 19 March 2020, various public health measures, including limits on both in-person outdoor and indoor gatherings [4, 5], were put in place in Queensland. Further legislation on 23 March directed the closure of nonessential businesses [5]. Border restrictions were then implemented on 26 March so that anyone arriving from another state or territory was required to self-quarantine for 14 days [5].

It is well recognised that the importation of travel-associated NG is a key driver of both NG infections and associated AMR in Australia. For example, a recent study showed that 44% of infections were acquired via overseas travel or contact with an overseas traveller [6]. Therefore, it would seem counterintuitive for rates to have increased during this period. To better understand this, we utilised an NG single nucleotide polymorphism (SNP)-based genotyping tool to examine gonococcal strains for the first 6 months of 2020, with 1 January 2020 to 31 March 2020 classified as pre-COVID-19 restrictions (pre-restrictions) and 1 April 2020 to 30 June 2020 classified as post-COVID-19 restrictions (post-restrictions). The date breakdown is based on the date of specimen collection.

A total of 800 NG isolates from individual patients (one isolate per patient) from 1 January to 31 March (pre-restrictions) and from 1 April to 30 June 2020 (post-restrictions) in Queensland were examined. Approximately 70% of isolates (cultures) referred to us are from Sexual Health clinics and the remainder from general practitioners (GPs). These isolates were estimated to comprise 92.03% of all gonococci isolated by culture during this period [2, 3]. Isolate nucleic acid extracts were genotyped using previously described iPLEX genotyping methods targeting 14 informative SNPs within NG housekeeping genes, as well as 12 chromosomal mutations reported to contribute to NG AMR [7].

Of the 800 isolates, 763 (95%) were successfully genotyped and the remaining 37 isolates that failed to be genotyped were excluded from further analysis. Overall, 71 unique NG genotypes were observed (detailed in Supplementary Table S2); 31 genotypes appeared in both pre- and post-restrictions (total = 707 isolates; pre-restrictions *n* = 413 isolates; post-restrictions *n* = 294 isolates), 34 genotypes appeared only in the pre-restriction time period (*n* = 50 isolates), and 6 genotypes were only observed post-restrictions (*n* = 6 isolates). Figure 1 illustrates the top 25 genotypes circulating in Queensland in the first 6 months of 2020: G1–G25 comprising the most common genotypes (four or more isolates each) and the remaining genotypes G26–G71 comprising three or fewer isolates. There was no significant reduction in the most common NG genotypes after COVID-19 restrictions eased in Queensland, and in fact, some genotypes proportionally increased (using Fisher’s exact test). These included several genotypes (G2, G3, G5, G6, G10, G12, G13, G15, G16, G20, and G24) with G2, G6, and G13 all exhibiting significant increases post-restrictions; *p* < 0.05. These genotypes were associated with both heterosexual (G2) and MSM networks (G6 and G13), based on male/female proportion (Figure 1a). G2 was observed in both the major metropolitan area of Southeast Queensland (SEQ) and regional areas of North Queensland (NQ); G6 in SEQ, NQ, and Far NQ; and G13 in SEQ (Figure 1b).

**Figure 1.**
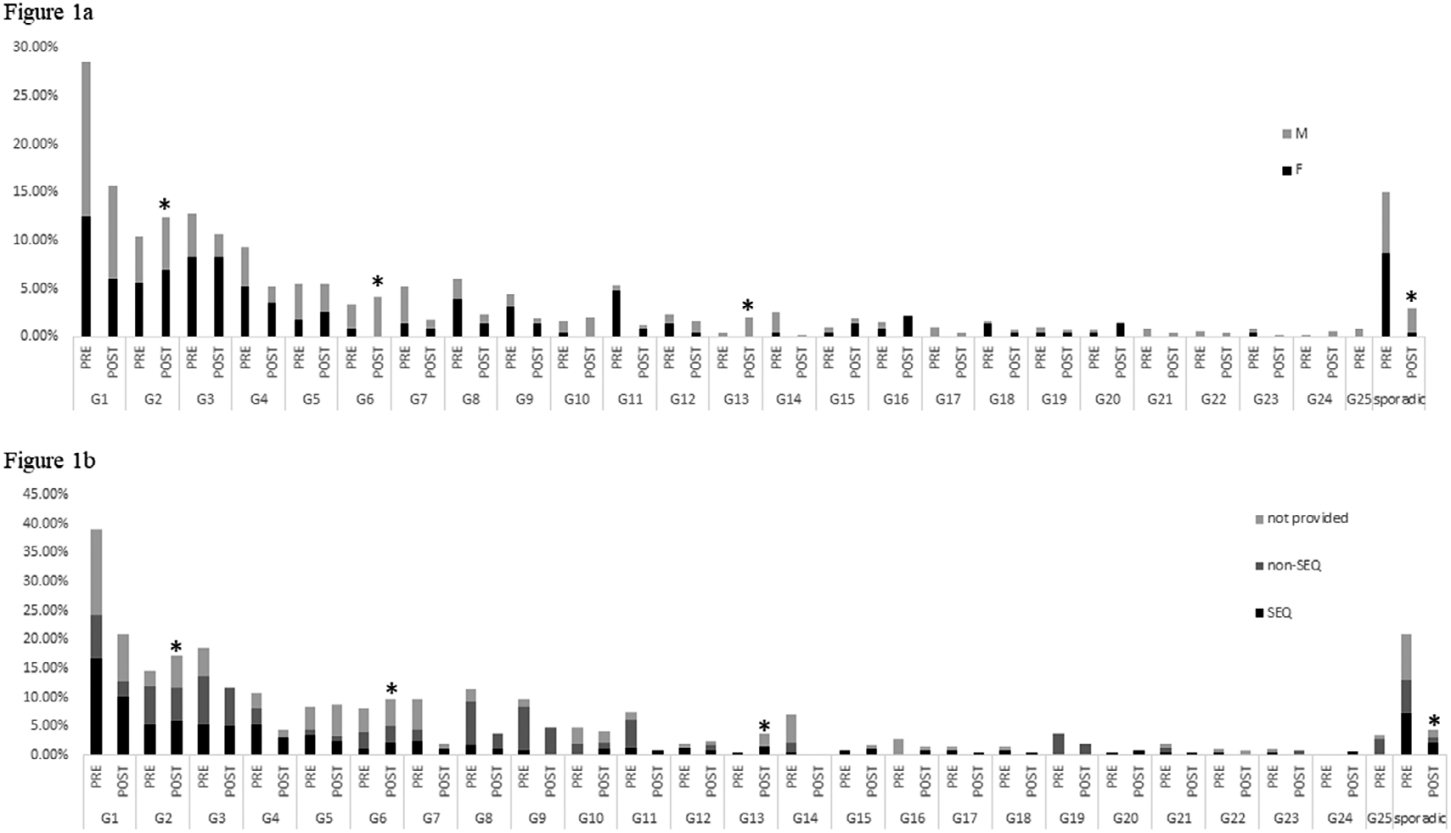
Proportion of the most common genotypes in Queensland pre and post COVID-19 restrictions for the first 6 months of 2020. Graph (a) represents the female and male proportions for each genotype and graph (b) represents the regional proportions for each genotype. Abbreviations: not provided, isolates without regional data; SEQ, South East Queensland

In contrast, there was a significant difference in prevalence for the less-common genotypes (G26–G71) between pre- and post-restriction periods (*p* < 0.05), with a marked reduction in these genotypes (comprising three or fewer isolates) for both males and females (Figure 1a), across both SEQ and non-SEQ regions (Figure 1b). Notably, there were 46 of the lesser common genotypes in total; of these, 7 were observed in both the pre- and post-restriction periods, whereas 33 were detected in the pre-restriction period, and 6 were detected in the post-restriction period only. This suggests, consistent with the border closures, that the diversity of circulating genotypes relies on both interstate and overseas incursions.

These results show that the public health restrictions for the mandated approximately 10-week period in late March 2020 were not sufficient to affect rates of infection or reduce the prevalence of well-established genotypes in our population. In fact, based on these data, the prevalence of certain NG genotypes significantly increased. While the reasons for these increases are unclear, we can postulate that these were due to no change in sexual relationships with a more limited pool of sexual partners enhancing the proportions of already circulating genotype. Alternatively, this may also suggest that condomless sex escalated during this period and/or access to sexual health services decreased. We postulate that health-seeking behaviours may have also changed during this period of increased restrictions, resulting in non-timely diagnosis and treatment of infection. This highlights the need to consider the implications of a pandemic and subsequent restrictions on other infections.

Limitations-wise, a longer-term study may be warranted along with sexual orientation data for increased precision of sexual networks. Yet overall, based on available data, the only obvious positive impact of the restrictions in terms of gonorrhoea was a reduction in the introduction of novel strains into our population, which potentially reduced the incursion of AMR strains. However, this may only be a reprieve. Notably, FC428, previously detected in Australia [8] and not observed in Queensland in 2020, is now known to be circulating widely in Asia [9, 10]. Such new AMR threats highlight that further enhancing surveillance concerning AMR and associated AMR molecular targets in NG will be valuable as restrictions on local and international travel further ease.

## Data Availability

Data will be made available upon request with the corresponding author.

## Supplementary material

For supplementary material accompanying this paper visit https://doi.org/10.1017/S0950268823000523. click here to view supplementary material
