# Supplementary material for: Decreased Neisseria gonorrhoeae genotypic diversity following COVID-19 restrictions in Queensland, Australia 2020
Source: Epidemiol Infect. 2023 Apr 13;151:e67. doi: 10.1017/S0950268823000523 (PMC10154642; doi:10.1017/S0950268823000523)
Supplement: Supplementary file 1 [file S0950268823000523sup001.docx]

**Title:** Increased *Neisseria gonorrhoeae* infection rates with decreased genotypic diversity, following COVID-19 restrictions in Queensland, Australia 2020.

**Journal name:** European Journal of Clinical Microbiology & Infectious Diseases

**Authors:**

Ella Trembizki ^1^_,_ Bushra Alharbi ^1, 2^, Amy V Jennison ^3^, Vicki Hicks ^3^, Emma Sweeney^1^, David Whiley ^1, 4^.

*^1^The University of Queensland Centre for Clinical Research, Faculty of Medicine, The University of Queensland - Brisbane (Australia),*

*^2^Taibah University, Faculty of Pharmacy - Madinah (Saudi Arabia),*

*^3^Public Health Microbiology, Queensland Health Forensic and Scientific Services - Brisbane (Australia),*

*^4^Pathology Queensland Central Laboratory - Brisbane (Australia)*

**Corresponding author details:**

Ella Trembizki

Address: University of Queensland, UQ Centre for Clinical Research

Building 71/918, Royal Brisbane and Women’s Hospital

QLD 4029

Email: [e.trembizki@uq.edu.au](about:blank)

| Supplementary Table 1. 2016 - 2020 quarterly Queensland gonorrhoea notification data | | | | | |
| --- | --- | --- | --- | --- | --- |
|  | **2016** | **2017** | **2018** | **2019** | **2020** |
| Q1 | 854 | 1431 | 1200 | 1465 | 1852 |
| Q2 | 927 | 1280 | 1112 | 1351 | 1533 |
| Q3 | 1058 | 1156 | 1146 | 1511 | 1568 |
| Q4 | 1133 | 1132 | 1395 | 1579 | 1312 |


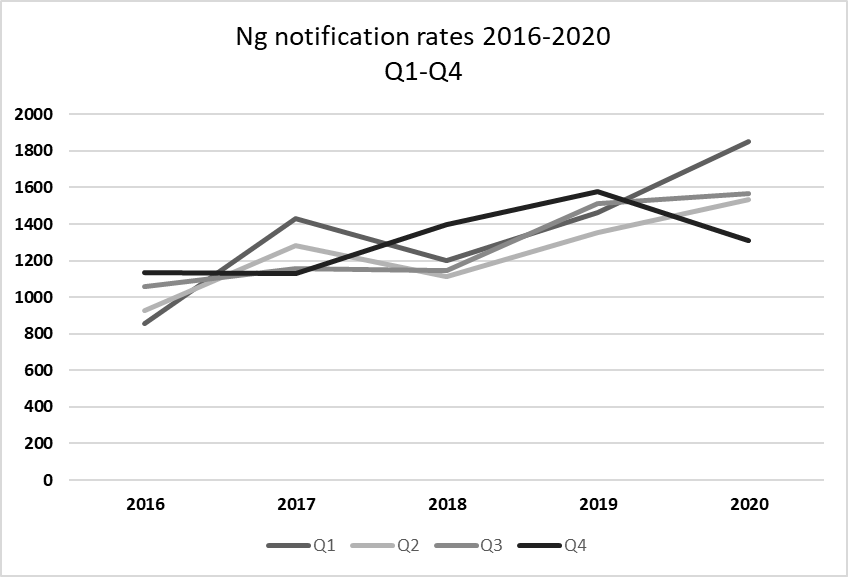


**Supplementary Figure 1. NG notification rates 2016-2020 Q1-Q4.** This graph demonstrates quarterly notifications remain steady throughout the years with a subtle increase in 2020.

**Supplementary Table 2. Data summary of all NG isolates Pre (n=463) and Post (n=300) covid-19 restrictions included in the study and their molecular and phenotypic AMR data**

|  | | | | | | | | | ^b^AMR mutation marker | | | | | | | | | | | ^l^AMR phenotype  (Bacterial culture) | | | |
| --- | --- | --- | --- | --- | --- | --- | --- | --- | --- | --- | --- | --- | --- | --- | --- | --- | --- | --- | --- | --- | --- | --- | --- |
| Genotype | **PRE** | **POST** | **Significance** | **F** | **M** | **^a^SEQ** | **Non-SEQ** | **Not provided** | **^c^23S rRNA nucleotide 2059** | **^c^23S rRNA nucleotide 2611** | **^d^GyrA amino acid 91** | **^d^GyrA amino acid 95** | **^e^ponA1** | **^f^*mtrR* promoter A deletion?** | **^g^Menigicoccal *mtrR*** | **^h^*mtrR* promoter T insertion?** | **^i^MtrR amino acid 45** | **^j^PBP2 amino acid 501** | **^k^Mosaic PBP2?** | **Penicillin** | **Ceftriaxone**  **c** | **Ciprofloxacin** | **Azithromycin** |
| 1 | 114 | 65 | Not significant | 43 | 136 | 133 | 11 | 34 | WT | WT | WT | WT | L421P | G | WT | C | WT | WT | WT | LS | S | S | S |
| 2 | 38 | 45 | Significant at *p* <0.05 | 29 | 54 | 56 | 13 | 12 | WT | WT | WT | WT | WT | A | WT | T | WT | WT | WT | LS | S | S | S |
| 3 | 43 | 32 | Not significant | 38 | 37 | 51 | 16 | 7 | WT | WT | WT | WT | WT | A | WT | C | WT | WT | WT | LS | S | S | S |
| 4 | 34 | 17 | Not significant | 20 | 31 | 3 | 6 | 1 | WT | WT | WT | WT | L421P | G | WT | C | WT | WT | WT | LS | S | S | S |
| 5 | 24 | 21 | Not significant | 10 | 35 | 29 | 2 | 14 | WT | WT | S91F | D95G | L421P | G | WT | C | WT | A501T | WT | LS | S | R | S |
| 6 | 15 | 22 | Significant at *p* < .05 | 2 | 35 | 17 | 6 | 13 | WT | WT | S91F | D95A | WT | A | WT | C | WT | WT | WT | LS | S | R | S |
| 7 | 24 | 7 | Not significant | 5 | 26 | 18 | 2 | 9 | WT | WT | WT | WT | WT | A | WT | C | WT | WT | WT | LS | S | S | S |
| 8 | 20 | 8 | Not significant | 12 | 16 | 14 | 11 | 3 | WT | WT | S91F | D95A | WT | A | WT | C | WT | WT | WT | PPNG | S | R | S |
| 9 | 14 | 6 | Not significant | 10 | 10 | 5 | 13 | 2 | WT | WT | WT | WT | WT | A | WT | C | WT | WT | WT | PPNG | S | S | S |
| 10 | 7 | 11 | Not significant | 1 | 17 | 7 | 3 | 7 | WT | WT | WT | WT | WT | A | WT | C | WT | WT | WT | LS | S | S | S |
| 11 | 14 | 4 | Not significant | 13 | 5 | 11 | 5 | 2 | WT | WT | WT | WT | WT | A | WT | C | WT | WT | WT | LS | S | S | S |
| 12 | 8 | 7 | Not significant | 4 | 11 | 11 | 1 | 2 | WT | WT | WT | WT | WT | A | WT | C | WT | WT | WT | PPNG | S | S | S |
| 13 | 2 | 11 | Significant at *p* < .05 | - | 13 | 10 | - | 3 | WT | WT | S91F | D95A | L421P | A | Mut | C | WT | WT | WT | LS | S | R | S |
| 14 | 12 | 1 | Significant at *p* < .05 | 1 | 12 | 3 | 2 | 7 | WT | WT | S91F | D95A | L421P | A | WT | C | WT | WT | WT | LS | S | R | S |
| 15 | 4 | 6 | Not significant | 4 | 6 | 9 | - | 1 | WT | WT | S91F | D95A | WT | A | WT | C | WT | WT | Mosaic | PPNG | S | R | S |
| 16 | 5 | 5 | Not significant | 7 | 3 | 5 | - | 5 | WT | Mut | WT | WT | WT | A | WT | C | WT | WT | WT | LS | S | S | R |
| 17 | 5 | 2 | Not significant | - | 7 | 6 | - | 1 | WT | WT | S91F | D95A | L421P | A | WT | C | WT | WT | WT | LS | S | R | S |
| 18 | 5 | 2 | Not significant | 4 | 3 | 6 | - | 1 | WT | WT | S91F | D95G | L421P | G | WT | C | G45D | A501V | WT | LS | S | R | S |
| 19 | 4 | 2 | Not significant | 2 | 4 | - | 6 | - | WT | WT | WT | WT | L421P | A | WT | C | WT | WT | WT | LS | S | S | S |
| 20 | 2 | 4 | Not significant | 4 | 2 | 6 | - | - | WT | WT | S91F | D95A | WT | A | WT | C | WT | WT | WT | PPNG | S | R | S |
| 21 | 4 | 2 | Not significant | - | 6 | 4 | 1 | 1 | WT | WT | WT | WT | WT | A | WT | C | WT | WT | WT | LS | S | S | S |
| 22 | 3 | 2 | Not significant | - | 5 | 3 | - | 2 | WT | WT | S91F | D95A | WT | A | WT | C | WT | WT | Mosaic | PPNG | S | R | S |
| 23 | 3 | 1 | Not significant | 1 | 3 | 2 | 1 | 1 | WT | WT | S91F | D95A | WT | A | WT | C | WT | WT | WT | PPNG | S | R | S |
| 24 | 1 | 3 | Not significant | - | 4 | 4 | - | - | WT | WT | S91F | D95A | L421P | A | WT | C | WT | WT | WT | PPNG | S | R | S |
| 25 | 4 | 0 | Not significant | - | 4 | - | 3 | 1 | WT | WT | S91F | D95A | WT | A | WT | C | WT | A501V | WT | PPNG | S | R | S |
| 26 | 2 | 1 | Not significant | - | 3 | 2 | - | 1 | WT | WT | WT | WT | WT | A | WT | C | WT | WT | WT | LS | S | S | S |
| 27 | 3 | 0 | Not significant | 1 | 2 | 3 | - | - | WT | WT | WT | WT | L421P | A | WT | C | WT | WT | WT | PPNG | S | S | S |
| 28 | 1 | 2 | Not significant | - | 3 | 3 | - | - | WT | WT | S91F | D95A | L421P | A | WT | C | WT | WT | WT | LS | S | R | S |
| 29 | 1 | 1 | Not significant | 1 | 1 | 2 | - | - | WT | WT | WT | WT | WT | A | WT | C | WT | WT | WT | PPNG | S | S | S |
| 30 | 1 | 1 | Not significant | 1 | 1 | 1 | - | 1 | WT | WT | WT | WT | L421P | G | WT | C | WT | WT | WT | LS | S | S | S |
| 31 | 2 | 0 | Not significant | 2 | - | 1 | 1 | - | WT | Mut | WT | WT | WT | A | WT | C | G45D | WT | WT | LS | S | S | R |
| 32 | 2 | 0 | Not significant | 2 | - | 1 | 1 | - | WT | WT | WT | WT | WT | A | WT | C | WT | WT | WT | PPNG | S | S | S |
| 33 | 2 | 0 | Not significant | 1 | 1 | 1 | - | 1 | WT | WT | S91F | D95A | L421P | A | WT | C | WT | WT | WT | PPNG | S | R | S |
| 34 | 2 | 0 | Not significant | 2 | - | - | 1 | 1 | WT | WT | WT | WT | WT | A | WT | C | WT | WT | WT | LS | S | S | S |
| 35 | 1 | 1 | Not significant | - | 2 | 2 | - | - | WT | WT | S91F | D95G | L421P | G | WT | C | WT | WT | Mosaic | R | S | R | S |
| 36 | 2 | 0 | Not significant | - | 2 | 2 | - | - | WT | WT | S91F | D95G | L421P | G | WT | C | WT | WT | WT | LS | S | R | S |
| 37 | 2 | 0 | Not significant | 1 | 1 | 2 | - | - | WT | WT | S91F | D95A | WT | G | WT | C | WT | WT | WT | PPNG | S | R | S |
| 38 | 1 | 1 | Not significant | 1 | 1 | 1 | - | 1 | - | WT | S91F | D95G | L421P | G | WT | C | WT | A501T/WT | WT | LS | S | R | S |
| 39 | 1 | 1 | Not significant | - | 2 | 2 | - | - | WT | WT | WT | WT | WT | A | Mut | C | WT | WT | WT | LS | S | S | S |
| 40 | 2 | 0 | Not significant | - | 2 | - | - | 2 | WT | WT | S91F | D95A | WT | A | WT | C | WT | WT | WT | LS | S | R | S |
| 41 | 2 | 0 | Not significant | - | 2 | 2 | - | - | WT | WT | S91F | D95A | WT | A | WT | C | WT | WT | WT | PPNG | S | R | S |
| 42 | 2 | 0 | Not significant | - | 2 | 1 | - | 1 | WT | WT | WT | WT | WT | G | WT | C | WT | WT | WT | LS | S | S | S |
| 43 | 2 | 0 | Not significant | 1 | 1 | 2 | - | - | WT | WT | S91F | D95A | WT | A | Mut | C | WT | WT | WT | LS | S | R | S |
| 44 | 2 | 0 | Not significant | 1 | 1 | 1 | - | 1 | WT | WT | WT | WT | WT | G | WT | C | WT | WT | WT | LS | S | S | S |
| 45 | 1 | 0 | Not significant | 1 | - | - | 1 | - | WT | WT | WT | WT | WT | A | WT | C | WT | WT | WT | PPNG | S | S | S |
| 46 | 0 | 1 | Not significant | - | 1 | - | 1 | - | WT | WT | WT | WT | WT | A | WT | C | WT | WT | WT | PPNG | S | S | S |
| 47 | 0 | 1 | Not significant | - | 1 | 1 | - | - | WT | WT | WT | WT | WT | A | WT | C | WT | WT | WT | PPNG | S | S | S |
| 48 | 1 | 0 | Not significant | - | 1 | 1 | - | - | WT | WT | WT | WT | WT | A | WT | C | WT | WT | WT | LS | S | S | S |
| 49 | 1 | 0 | Not significant | - | 1 | 1 | - | - | WT | WT | WT | WT | WT | A | WT | C | WT | WT | WT | PPNG | S | S | S |
| 50 | 0 | 1 | Not significant | - | 1 | 1 | - | - | WT | WT | WT | WT | WT | A | WT | C | G45D | WT | WT | LS | S | R | S |
| 51 | 1 | 0 | Not significant | 1 | - | 1 | - | - | WT | WT | WT | WT | WT | A | WT | T | WT | WT | WT | LS | S | S | S |
| 52 | 1 | 0 | Not significant | 1 | - | - | - | - | WT | WT | WT | WT | WT | A | WT | C | WT | WT | WT | LS | S | S | S |
| 53 | 1 | 0 | Not significant | 1 | - | 1 | - | - | WT | WT | S91F | D95A | WT | A | WT | C | WT | WT | WT | PPNG | S | R | S |
| 54 | 1 | 0 | Not significant | 1 | - | 1 | - | - | WT | WT | WT | WT | WT | A | WT | C | WT | WT | WT | PPNG | S | S | S |
| 55 | 1 | 0 | Not significant | - | 1 | 1 | - | - | WT | WT | WT | WT | WT | A | WT | C | WT | WT | WT | LS | S | S | S |
| 56 | 0 | 1 | Not significant | - | 1 | - | - | 1 | WT | WT | WT | WT | L421P | G | WT | C | WT | WT | WT | LS | S | S | S |
| 57 | 1 | 0 | Not significant | 1 | - | 1 | - | - | WT | WT | S91F | WT | L421P | A | WT | C | WT | A501V | Mosaic | LS | S | R | S |
| 58 | 0 | 1 | Not significant | - | 1 | 1 | - | - | WT | WT | WT | WT | WT | A | WT | C | WT | WT | WT | PPNG | S | S | S |
| 59 | 1 | 0 | Not significant | 1 | - | - | - | 1 | WT | WT | S91F | WT | L421P | A | WT | C | WT | WT | Mosaic | R | S | R | S |
| 60 | 0 | 1 | Not significant | - | 1 | 1 | - | - | WT | WT | S91F | D95A | WT | A | WT | C | WT | A501V | WT | LS | S | R | S |
| 61 | 1 | 0 | Not significant | - | 1 | - | 1 | - | WT | WT | S91F | D95A | WT | A | WT | C | WT | WT | WT | PPNG | S | R | S |
| 62 | 1 | 0 | Not significant | - | 1 | - | - | 1 | WT | WT | S91F | D95G | L421P | A | WT | C | WT | WT | WT | PPNG | S | R | S |
| 63 | 1 | 0 | Not significant | - | 1 | 1 | - | - | WT | WT | S91F | D95A | L421P | A | WT | C | WT | WT | WT | LS | S | R | S |
| 64 | 1 | 0 | Not significant | - | 1 | 1 | - | - | WT | WT | WT | WT | WT | G | WT | C | WT | WT | WT | LS | S | S | S |
| 65 | 1 | 0 | Not significant | - | 1 | 1 | - | - | WT | WT | WT | WT | WT | A | WT | C | WT | WT | WT | LS | S | S | S |
| 66 | 1 | 0 | Not significant | - | 1 | 1 | - | - | G | WT | WT | WT | WT | A | Mut | C | WT | WT | WT | LS | S | S | R |
| 67 | 1 | 0 | Not significant | - | 1 | 1 | - | - | WT | WT | WT | WT | WT | A | Mut | C | WT | WT | WT | LS | S | R | S |
| 68 | 1 | 0 | Not significant | - | 1 | 1 | - | - | WT | WT | S91F | D95A | WT | A | WT | C | WT | WT | WT | PPNG | S | R | S |
| 69 | 1 | 0 | Not significant | - | 1 | 1 | - | - | WT | WT | S91F | D95G | L421P | A | WT | C | WT | WT | WT | PPNG | S | R | S |
| 70 | 1 | 0 | Not significant | - | 1 | - | - | 1 | WT | WT | S91F | D95A | L421P | A | WT | C | WT | WT | WT | LS | S | R | S |
| 71 | 1 | 0 | Not significant | - | 1 | - | - | 1 | WT | WT | S91F | D95G | L421P | G | WT | C | WT | A501T | WT | R | S | R | S |
| total | 463 | 300 | - | 231 | 532 | 598 | 106 | 148 |  |  |  |  |  |  |  |  |  |  |  |  |  |  |  |
| ^a^ SEQ: South East Queensland  ^b^ AMR marker (Antimicrobial resistance markers): genotyping using iPLEX-AMR, targeting 11 chromosomal mutations informative of NG AMR (1).  ^c^23 rRNA: 23S rRNA C2611T and A2059G mutations associated with macrolide resistance and high-level resistance, respectively.  ^d^GyrA amino acid 91 and 95 substitutions: *gyrA* mutations associated with ciprofloxacin resistance..  ^e^*ponA* [penicillin-binding protein1 (PBP1)]: The alteration of amino acid at position 421 is associated with penicillin increased MICs.  ^f^*mtrR* promoter A deletion (adenine-deletion i*n the mtrR* gene that encodes a transcriptional repressor of the *mtrCDE* multidrug efflux transporter genes), ^g^menigicoccal-*mtrR* (meningococcal *mtrR* sequence in NG strain), ^h^*mtrR* promoter T insertion (mtrR thymine-insertion), ^i^MtrR amino acid 45 (Gly-45 (G)-to-Asp-45 (D) replacement in MtrR protien): associated with reduced susceptibility to azithromycin and penicillin.  ^j^PBP2 (Penicillin Binding Protein 2, coded by *penA*) substitutions at 501 position: Associated with NG decreased susceptibility to ceftriaxone.  ^k^Mosaic PBP2 (Mosaic-like structure of *penA* gene): Associated with NG decreased susceptibility to ceftriaxone and cefixime.  ^l^ AMR phenotype: represents the AMR profile held by the majority of isolates for each genotype  Susceptibility categories are defined for antibiotics tested as follows: For penicillin: S = sensitive (MIC ≤ 0.03 mg/L), LS = less sensitive (MIC 0.06–0.5 mg/L), R = resistant (MIC ≥1 mg/L), PPNG = penicillinase-producing *N. gonorrhoeae;* For ceftriaxone: S = sensitive (MIC ≤ 0.03 mg/L), LS = less sensitive (MIC 0.06–0.125 mg/L)*;* For ciprofloxacin: S = sensitive (MIC ≤ 0.03 mg/L), R=resistant (MIC ≥1 mg/L)*;* For azithromycin: S = sensitive (MIC ≤ 0.5 mg/L), R = resistant (MIC ≥1 mg/L) | | | | | | | | | | | | | | | | | | | | | | | |

**References**

1. Trembizki E, Wand H, Donovan B, Chen M, Fairley CK, Freeman K, et al. The molecular epidemiology and antimicrobial resistance of Neisseria gonorrhoeae in Australia: a nationwide cross-sectional study, 2012. 2016;63(12):1591-8.
